# Content Refinement and Multicenter Validation of the ELIGES Patient Experience Questionnaire Focused on Nursing Care During Hospitalization: Study Protocol

**DOI:** 10.3390/nursrep16090331

**Published:** 2026-09-09

**Authors:** Noelia Navamuel-Castillo, Josep-Oriol Casanovas-Marsal, Ángel Boned-Galán, Ana Belén Hernández-Ruiz, Nieves López-Ibort, Ana Gascón-Catalán

**Affiliations:** 1 Hospital Universitario Miguel Servet de Zaragoza, 50009 Zaragoza, Spain; nnavamuel@salud.aragon.es (N.N.-C.); acboned@salud.aragon.es (Á.B.-G.); 2 Instituto de Investigación Sanitaria de Aragón, 50009 Zaragoza, Spain; jocasanovas@iisaragon.es; 3 Hospital Universitario Lozano Blesa, 50009 Zaragoza, Spain; abhernandezr@salud.aragon.es (A.B.H.-R.); nlopezi@salud.aragon.es (N.L.-I.); 4 Departamento de Fisiatría y Enfermería, Facultad de Ciencias de la Salud. Universidad de Zaragoza, 50009 Zaragoza, Spain

**Keywords:** patient-centered care, patient preferences, quality of health care, surveys and questionnaires, validation study, hospital units, hospitalization, nursing, nurses

## Abstract

**Background:** Patient experience has become a key component of healthcare quality assessment and improvement. Although its importance is increasingly recognized, there is a lack of standardized instruments specifically designed to assess patient experience in relation to nursing care during hospitalization. The starting point for this study is the ELIGES-Patient Experience questionnaire developed in 2024. This study aims to refine its content by incorporating the patient perspective and to evaluate its psychometric properties through a multicenter study. The final questionnaire will be intended to assess patient experience in relation to nursing care in inpatient units, providing a reliable tool for identifying opportunities for improvement and supporting person-centered care. **Methods:** A three-phase multicenter mixed-methods study will be conducted. First, a focus groups with expert patients will identify the key dimensions of patient experience related to nursing care during hospitalization. The initial questionnaire will then be reviewed and revised, yielding a first provisional version. Second, a national Delphi study involving expert patients will establish consensus on the questionnaire content, thereby yielding a consensus-based provisional version of the original instrument. Third, this provisional version of the questionnaire will undergo multicenter psychometric validation in six Spanish public hospitals. A pilot study will assess temporal stability and agreement, followed by administration of the provisional version to 2271 hospitalized patients to evaluate its psychometric properties. **Expected Results:** Following content refinement and psychometric validation, the resulting instrument is expected to provide a standardized measure of hospitalized patients’ experience of nursing care that may help identify areas for future evaluation and quality-improvement initiatives. **Conclusions:** Patient experience measurement lacks standardization, which hinders comparability and limits its use as a quality improvement tool. This highlights the need for instruments that are conceptually sound, methodologically robust, and operationally applicable. A distinctive feature of this protocol is the active involvement of patients throughout the entire process, in keeping with the principles of person-centered care and helping to strengthen the relationship between healthcare professionals and patients.

## 1. Introduction

In the current healthcare context, patient experience has become a fundamental component of healthcare quality assessment and continuous improvement and is regarded as one of the three core pillars of healthcare quality, together with patient safety and clinical effectiveness [1,2,3,4]. Evidence suggests that these elements act synergistically to ensure safe, effective, and genuinely patient-centered care [5].

Since 1966, when Avedis Donabedian introduced the concept of the “patient voice” as a key indicator of healthcare quality, patient experience has evolved into a critical indicator of quality of care, guiding the transition toward patient-centered care that is respectful of and responsive to individual preferences, needs, and values [6,7].

Within this framework, the World Health Organization (WHO) stated in 2007 that people-centered care is a core competency for all healthcare professionals and identified it as a fundamental component of quality care [3]. In this context, the WHO published People-Centered Health Care: A Policy Framework in 2007. This document emphasizes this approach in health policy and guides the design of more humane and integrated health systems [8]. Subsequently, in 2017, the WHO released the Framework on Integrated People-Centered Health Services, consolidating this perspective and describing a formal global framework intended to guide the transformation of health systems worldwide by positioning health service users as both a key element in this transformation and the focus of health system activity [9].

Several countries have progressively increased the prominence of the patient voice within their health system models. This has been reflected in the design of programs and models that promote the integration of patient experience across different levels of care. In the United States, the Patient-Centered Medical Home (PCMH) model is being implemented [10,11], organizing primary care in a comprehensive and patient-centered manner. In Saudi Arabia, the national Saudi Vision 2030 strategy, launched in 2016, incorporates patient experience as a strategic pillar of health system transformation [12]. In Europe, the United Kingdom has developed policies and programs, such as the National Patient Survey Programme and the quality standards of the National Institute for Health and Care Excellence (NICE), to ensure the measurement and continuous improvement of patient experience and to promote care that is safe, effective, equitable, evidence-based, and person-centered [13].

Despite these advances, the use of information derived from patient experience remains a relatively immature field of study, with uncertainties regarding which information is most relevant for analysis, how it should be integrated, and, above all, how it can be used to improve healthcare quality [9,14,15]. Patient-centered care requires consideration of the relational and functional aspects of healthcare delivery, as well as structural aspects and the design of clinical processes. However, its measurement remains challenging because of the lack of standardized metrics and the limited direct involvement of service users in defining the most appropriate indicators [4,9].

In this context, patient experience is defined as the sum of all interactions that an individual has with the healthcare system, shaped by organizational culture and the behavior of healthcare professionals throughout the entire care process, from the initial communication to discharge [16]. This concept distinguishes patient experience from satisfaction: satisfaction reflects an individual’s subjective evaluation in relation to their initial expectations, whereas patient experience assesses whether specific relevant behaviors and actions occurred and how frequently they occurred during the care process [4,17].

Nursing teams are a key component of patient experience. In hospital care processes, they carry out approximately 70% of all interventions performed directly with patients and their caregivers and account for approximately 65% of the hospital workforce [18]. Nurses remain in continuous contact with patients 24 h a day, and their influence on care planning and delivery has a direct impact on patient safety, clinical effectiveness, and perceived quality of care [1,5,9], potentially helping to make hospital care processes less traumatic and painful [7].

Furthermore, evidence shows that positive patient experiences during care processes are associated with a lower incidence of complications, better treatment adherence, and improved clinical outcomes. These findings highlight the importance of understanding and measuring patient experience in the hospital setting in relation to nursing care [2,7,19,20]. The structured and systematic measurement of patient experience enables information to be collected quickly and efficiently to identify areas for improvement and ensure that care is centered on, and better aligned with, patients’ actual needs than when only the perspectives of professionals and organizations are considered [2,6,7,21].

Patient experience is therefore a multidimensional construct. The NHS Patient Experience Framework supports this concept and is based on a modified version of the Picker Institute’s principles of patient-centered care. It identifies eight relevant components of the care experience: respect for patients’ expressed values, preferences, and needs; coordination and integration of care; information, communication, and education; physical comfort; emotional support; involvement of family and friends; continuity of care; and access to care [22].

Various tools have been developed internationally to assess patient experience, although they have limitations with regard to the specific measurement of nursing care. In the United Kingdom, regularly administered NHS surveys and the aforementioned NICE standards provide information on aspects such as respect, coordination, and clinical effectiveness [13,23,24]. In the United States, the Consumer Assessment of Healthcare Providers and Systems (CAHPS) surveys assess experience in different settings and have become widely used as a reference for improving person-centered care. The Hospital Consumer Assessment of Healthcare Providers and Systems (HCAHPS), which focuses specifically on the hospital setting, was developed from these surveys [25,26,27].

Other examples of questionnaires widely used in the literature include the Picker Patient Experience Questionnaire, which has been applied in different settings and countries through adapted versions [28,29,30]; the Patient Perceptions of Integrated Care (PPIC) questionnaire, originally developed in the United States but validated in the European context [31,32,33]; and the instrument developed by the European Task Force on Patient Evaluations of General Practice Care (EUROPEP), created in Europe [34] and subsequently translated and validated for use in different countries [35,36,37,38,39].

However, most of the questionnaires mentioned above, as well as the programs implemented in the countries described and in other countries that have adopted them, are based on healthcare quality assessment systems focused on patients’ overall perceptions rather than on a specific care context. This makes it difficult to identify specific areas for improvement within the services evaluated, particularly in relation to specific professional groups or clinical situations [21].

Furthermore, the availability of specific data derived from validated tools enables comparisons over time, both internally and externally, across different centers and care settings, provided that the instrument has demonstrated adequate measurement properties in those settings. It also facilitates the identification of performance indicators and may help guide the development of policies and practices aimed at continuous improvement, thereby strengthening a patient-centered organizational culture and facilitating the implementation of integrated strategies [6,7,14,21,30,40,41,42].

In this context, López-Ibort et al. developed the ELIGES-Patient Experience questionnaire in 2024, specifically to assess patient experience in relation to nursing care during hospitalization [43]. Its development used the previously described components of the NHS Patient Experience Framework as its conceptual framework [22]. During its initial validation, the instrument items were empirically organized into five dimensions (interrelationships, nursing care, information during hospitalization, information about patient rights, and discharge information), and two additional pain-related items were included [43].

Both the components defined in the NHS Patient Experience Framework and the dimensional structure identified during the initial validation of the ELIGES questionnaire constitute the conceptual and empirical starting points for the present study, the questionnaire revision process, and the subsequent psychometric evaluation of the instrument, without presupposing that its final dimensional structure must remain unchanged.

It is important to note that the development of the initial 2024 ELIGES-Patient Experience questionnaire was based primarily on the literature, a review of the Picker Patient Experience Questionnaire, and the involvement of expert professionals. After its development, a pilot study involving patients was conducted to assess aspects related to item comprehensibility, clarity, and relevance. The initial psychometric validation was subsequently conducted in a single hospital [43]. The purpose of this new study is to examine the content validity of the instrument in greater depth through more systematic incorporation of the patient perspective and to extend the evaluation of its psychometric properties across different hospital settings in several regions of Spain.

## 2. Methods

### 2.1. Objectives

Primary objective:

- To refine and validate the ELIGES-Patient Experience questionnaire, previously developed by López-Ibort et al. [43] to measure patient experience in relation to nursing care during hospitalization.

Secondary objectives:

- To identify and review, with the involvement of expert patients, the content considered relevant to patient experience in relation to nursing care during hospitalization, assessing its correspondence with the original instrument and the need to reword, add, or remove items.
- To reach consensus among expert patients on the relevance and appropriateness of the content to be included in the provisional version of the questionnaire.
- To evaluate the psychometric properties of the refined version of the provisional ELIGES questionnaire through a multicenter study conducted in six Spanish public hospitals.

### 2.2. Study Design

A sequential process of content refinement and multicenter psychometric validation will be undertaken of the ELIGES-Patient Experience questionnaire developed by López-Ibort et al. [43].

A multicenter study with a sequential design will be conducted. It will comprise three consecutive and distinct phases: qualitative content review and revision involving expert patients; consensus on the content through a Delphi process involving expert patients; and evaluation of the psychometric properties of the refined version of the instrument through a multicenter study.

The study will follow the COSMIN (COnsensus-based Standards for the selection of health Measurement INstruments) recommendations for evaluating the measurement properties applicable to the study design [44]. Specifically, content validity will be addressed by considering the relevance, comprehensiveness, and comprehensibility of the items through iterative content review and consensus-building involving expert patients. Structural validity and internal consistency will subsequently be evaluated. Temporal stability and agreement between repeated administrations will also be examined. Aspects related to the interpretability of the instrument, such as response distributions and potential floor and ceiling effects, will also be considered. Measurement properties that are not part of the design of the present protocol, such as responsiveness, will not be evaluated. The specific procedures and criteria for each property are described in the different study phases and in the statistical analysis section.

Six public hospitals from different regions of Spain will participate in the multicenter psychometric evaluation phase. Hospitals from different geographic and organizational settings were contacted in an effort to incorporate diversity of contexts. The centers ultimately included were those that agreed to participate and collaborate in the project. This selection increases the geographic and organizational heterogeneity of the study and enables the instrument to be evaluated in different hospital settings, without claiming national representativeness. The participating hospitals will be Hospital El Bierzo (Ponferrada, Castile and León), Hospital Universitari i Politècnic La Fe (Valencia, Valencian Community), Hospital Universitario La Paz (Madrid, Community of Madrid), Hospital Clínico Universitario Lozano Blesa (Zaragoza, Aragon), Hospital Universitario Miguel Servet (Zaragoza, Aragon), and Hospital Universitari Sant Joan de Reus (Reus, Catalonia).

#### 2.2.1. Phase 1. Focus Group with Patient Associations

*Design*

**Figure 1:**
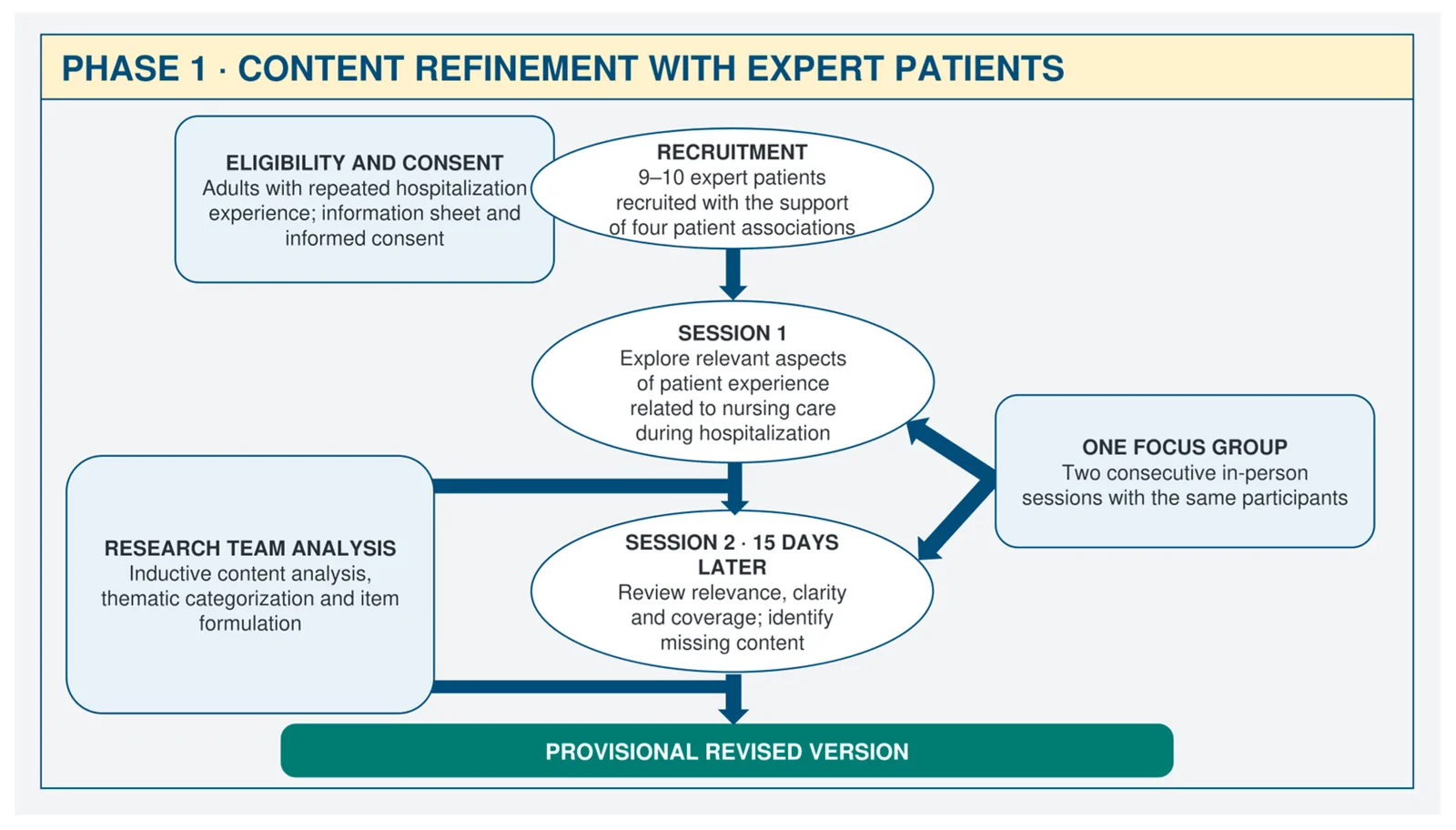
Diagram illustrating the first phase: focus groups with expert patients. Arrows indicate show the flow of the study procedures.

A qualitative research technique involving a focus group with expert patients will be used (Figure 1). This focus group will serve to identify, directly from the perspective of expert patients, the key elements of patient experience during hospitalization that are related to nursing care.

The focus group will be used as an exploratory technique to generate qualitative data, with interaction among participants serving as a mechanism for identifying the key elements of patient experience during hospitalization and their relationship with nursing care. This information will be used to complement the initial questionnaire and inform any necessary revisions.

*Sample*/*Participants*

Expert patients will participate through the Spanish Association Against Cancer (AECC), the National Federation of Associations for the Fight Against Kidney Diseases (ALCER), the Aragon Patient Forum, and the Association of Aragonese Women with Genital and Breast Cancer (AMACGEMA).

An expert patient will be defined as an individual who becomes aware that they are primarily responsible for their own health and who has the tools, motivation, and confidence needed to fulfill this role, always in close collaboration with healthcare professionals [45].

Inclusion criteria: being aged 18 years or older, meeting the established criteria for being considered an expert patient [45], having been hospitalized on more than one occasion during their lifetime, and being available to attend the scheduled sessions in person.

A total of 9–10 participants will be selected through criterion-based purposive sampling. To promote diversity of perspectives, efforts will be made to achieve a balanced composition by sex and to include participants of different ages who have experienced hospitalization in different care units and for different reasons.

The purpose of this group of participants is to provide diverse perspectives to inform the content review and revision process, rather than to be statistically representative of the hospitalized adult population.

The planned group size falls within the ranges recommended for this qualitative technique and is intended to balance diversity of perspectives with the opportunity for all participants to contribute and elaborate on their experiences during the sessions. A smaller number could limit the variety of perspectives and interactions generated, whereas larger groups may hinder balanced participation and reduce the time available to explore individual contributions in depth [46].

A single focus group will be conducted over two consecutive in-person sessions with the same participants attending both sessions. The sufficiency of the information obtained will be assessed according to the principle of information power proposed by Malterud et al. [47], whereby the adequacy of a qualitative sample depends, among other factors, on the specificity of the study aim, the relevance of the participants in providing information about the phenomenon under study, and the quality of the dialogue generated. In this study, the aim of the qualitative phase is specifically limited to reviewing and revising the content of a previously developed instrument.

Participants will be contacted to explain the entire procedure in detail and arrange the first session. Before the first meeting, they will receive a participant information sheet and an informed consent form, which must be signed and returned before the meeting takes place.

*Data Collection and Analysis*

The group will be moderated by a principal investigator with experience in qualitative research, with the assistance of two observers from the project research team. The sessions will be audio-recorded and transcribed verbatim.

During the first session, participants will be welcomed and the objectives of the meeting will be established and clarified. Informed consent will be obtained from each participant for study participation and for audio recording of each session.

The aim of the first session will be to explore the aspects of nursing care during hospital stays that patients consider most relevant and that may influence their experience of the hospitalization process.

Subsequently, the moderator and observers will independently and repeatedly review the transcripts. They will independently conduct an inductive content analysis, without using a predefined coding framework, followed by thematic categorization. The resulting codes and categories will be triangulated through consensus among the researchers. If discrepancies arise in the interpretation or classification of the content, the corresponding excerpts from the verbatim transcripts will be jointly reviewed until a consensus decision is reached. The resulting categories and codes will be operationalized as items for inclusion in the questionnaire. These items will be listed and integrated into a proposed item set for the final questionnaire. Redundant or ambiguous content will be excluded, and items containing more than one coded concept will be avoided. An audit trail will be maintained throughout this process, documenting the relationship between the identified codes and categories and each decision to add, reword, retain, or remove an item, together with the corresponding rationale.

This proposal will be reviewed at a second focus group meeting involving the same participants, held 15 days after the first. During this session, participants will assess the relevance, clarity, and appropriateness of the proposed items and will be explicitly asked whether each item is easy to understand or should be worded differently. They will also assess whether the items adequately represent relevant aspects of patient experience and whether any important content has not been addressed. Any comprehension difficulties, ambiguities, or wording problems identified will be used to revise the items before the Delphi phase.

The research team will subsequently transcribe the session and analyze the views collected, categorizing and classifying them according to each item under review. Based on this analysis, the initial ELIGES-Patient Experience questionnaire will be modified by identifying correspondences, gaps, and relevant new constructs. This process will yield the first provisional version of the questionnaire. The procedures conducted during this qualitative phase and their results will be reported in accordance with the applicable Consolidated Criteria for Reporting Qualitative Research (COREQ) proposed by Tong et al. [48].

#### 2.2.2. Phase 2. Delphi Method with a Panel of Expert Patients Recruited Through the Participating Associations

*Design*

**Figure 2:**
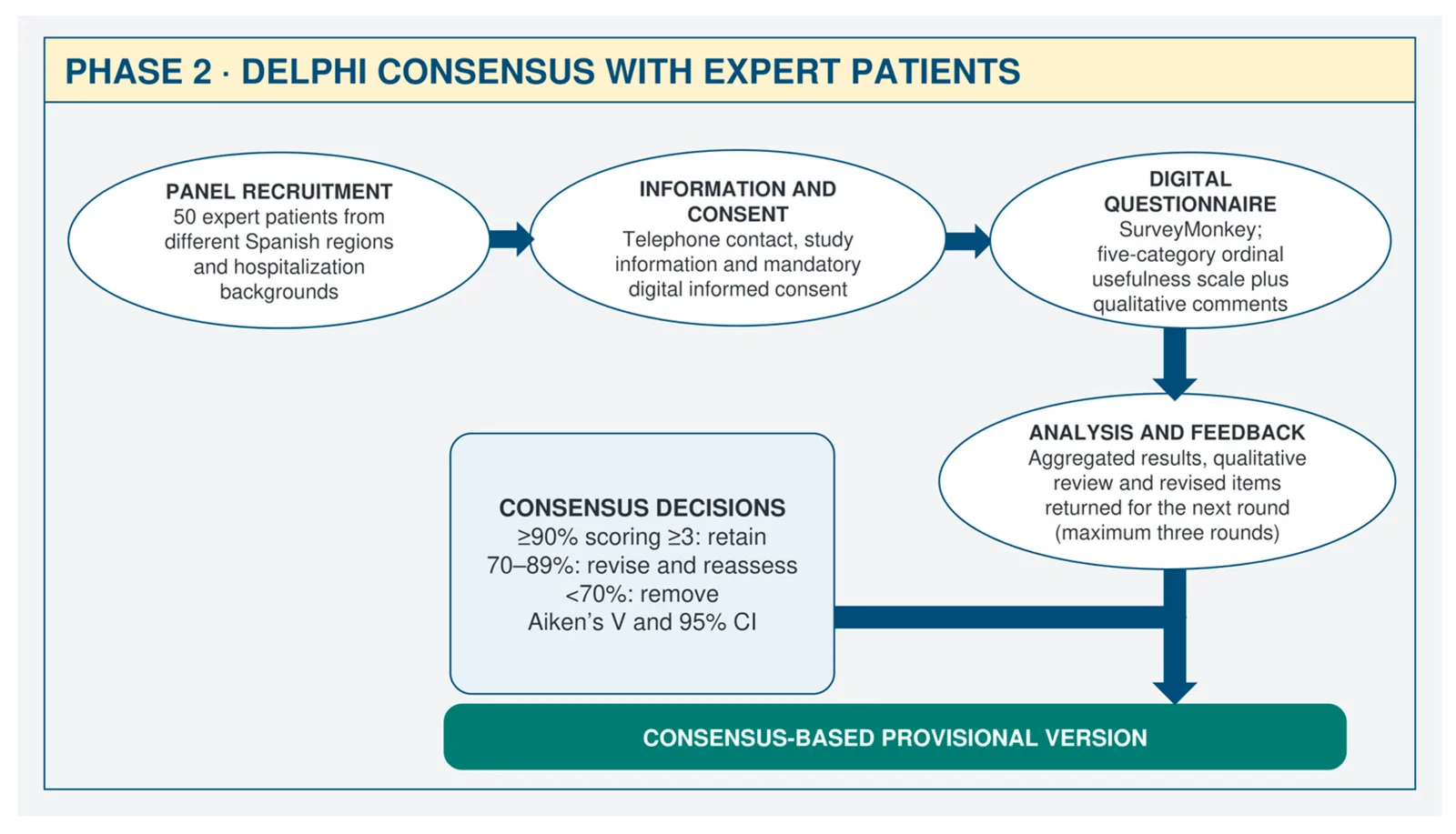
Diagram illustrating the second phase: Delphi study. Arrows show the Delphi flow.

A qualitative, prospective study involving a systematic and iterative process using the Delphi method [49] (Figure 2).

In this second qualitative phase, the Delphi technique [49] will be used as a structured consensus method to validate the content of the questionnaire revised in Phase 1. The relevance and usefulness of the included items, as well as their wording and clarity, will be considered. This technique enables the opinions of a panel of expert patients to be collected systematically, minimizing the influence of group dynamics and ensuring individual reflection in each round.

*Sample*/*Participants*

Fifty expert patients from across Spain will participate with the collaboration of patient associations.

Inclusion criteria: The same general inclusion criteria established for the preceding focus group stage will apply: being aged 18 years or older, meeting the established criteria for being considered an expert patient [45], and having been hospitalized on more than one occasion during their lifetime. In addition, participants must have the necessary resources and ability to complete the questionnaires digitally and provide a valid email address to receive study information, successive questionnaire rounds, and the corresponding feedback.

To promote diversity within the panel of expert patients, efforts will be made to include expert patients of different ages and sexes, from different geographic areas of Spain, and with hospitalization experiences involving different care units and reasons for admission.

*Data Collection and Analysis*

All participants will be contacted by telephone by the research team. The study procedure and their participation will be explained in detail. All questions will be addressed, and the research team will remain available to respond to any additional queries throughout the study. Participants will receive an explanatory document describing the entire process, as well as the informed consent form, so that they can review it in advance. Acceptance of informed consent will be incorporated into the digital questionnaires sent to participants and will be mandatory before they can continue completing the questionnaire.

The Delphi consensus technique [49] will be applied by distributing a digital questionnaire over a maximum of three iterative rounds. In each round, participants will receive aggregated information on the results of the preceding round. The questionnaire will be administered through the SurveyMonkey digital platform (SurveyMonkey Inc., San Mateo, CA, USA). Participants will rate each item according to its usefulness for measuring hospitalized patient experience, using a five-point ordinal scale (1 = completely unnecessary, 2 = unnecessary, 3 = useful, 4 = very useful, and 5 = essential). They will also have a dedicated space in which to provide more detailed comments on the relevance, content, or wording of each item, or on any other aspect they consider pertinent.

After each round, the research team will analyze all responses and consider all comments provided. These comments will be reviewed individually by three members of the research team, followed by a joint discussion to develop a proposal for final modifications. This proposal, together with the results obtained in the preceding round, will be summarized in a conclusions report that will be shared with all participating patients, together with the access link for completing a new evaluation. This process will be repeated until consensus is reached or a maximum of three rounds has been completed.

Participant retention will be documented across Delphi rounds. In each round, consensus percentages for each item will be calculated using as the denominator the number of participants who provide a valid rating for that item in that round. Participants who do not respond to a given round will not be included in the denominator for that round. The number and proportion of participants completing each round will be reported and considered when interpreting the robustness of the consensus reached.

Consensus to retain an item will be considered to have been reached when at least 90% of participants assign it a score of ≥3 (useful, very useful, or essential). When this percentage is between 70% and 89%, the item will be considered eligible for modification and will be reformulated on the basis of the qualitative comments provided by participants, before being reassessed in the next round. Items with fewer than 70% of ratings ≥ 3 will be removed.

As a complementary measure of content validity, Aiken’s V will be calculated for each item [50], together with its 95% confidence interval, using the method proposed by Penfield and Giacobbi [51] and the panel’s ordinal ratings. A conservative criterion of V = 0.70 will be adopted for interpretation [52]. An Aiken’s V value < 0.70, or a value ≥ 0.70 with a lower 95% confidence limit < 0.70, will indicate that the item requires review. These criteria will not be used as automatic rules for item retention or removal. When Aiken’s V results are discordant with the Delphi consensus criteria or qualitative findings, the item will be reviewed and, when appropriate and a subsequent Delphi round remains, reworded and reassessed. If discordance persists in the final round, the final decision will follow the prespecified Delphi consensus criteria, with Aiken’s V and qualitative findings used to contextualize and document the decision.

This procedure will allow the instrument to be revised iteratively and will provide additional evidence of content validity.

Content validity will be evaluated by considering relevance, comprehensibility, and comprehensiveness as distinct but complementary components. Relevance will refer to whether each item adequately represents an important aspect of patient experience related to nursing care during hospitalization and will be assessed through the qualitative findings from Phase 1 and the ratings and comments provided during the Delphi rounds. Comprehensibility will refer to the clarity, ease of understanding, ambiguity, and wording of each item and will be assessed through participants’ qualitative feedback in both phases. Comprehensiveness will refer to whether the item set adequately covers all relevant aspects of the construct; participants will therefore be asked to identify any important aspects of patient experience that are not represented in the questionnaire.

Qualitative findings from the focus group, Delphi ratings and comments, and Aiken’s V results will be considered jointly when deciding whether an item should be retained, reworded, added, or removed. No single source of evidence will be used in isolation to determine the final content of the questionnaire.

Item comprehensibility will be assessed through the responses and contributions provided by participants during Phases 1 and 2. When comprehension difficulties, ambiguities, or wording problems are reported, the research team will review the item and, when appropriate, reformulate it before it is reassessed in the next round.

All changes made, together with any explanatory comments agreed upon by the researchers, will be documented and communicated to participants in the conclusions report before each round.

Items retained in the questionnaire after the Delphi phase will constitute the consensus-based provisional version of the questionnaire, which will undergo psychometric validation in Phase 3. The number of items in this version will depend on the content review and consensus process conducted in Phases 1 and 2. The response format of the original ELIGES-Patient Experience questionnaire will be retained; this consists of a five-category ordinal frequency scale (always, almost always, sometimes, almost never, and never). This response scale should be distinguished from the ordinal usefulness scale proposed for use by the panel of expert patients during the Delphi consensus phase.

All items will be positively worded; therefore, reverse scoring will not be required.

#### 2.2.3. Phase 3. Multicenter Psychometric Validation of the Provisional Questionnaire on Patient Experience in Relation to Nursing Care

*Design*

**Figure 3:**
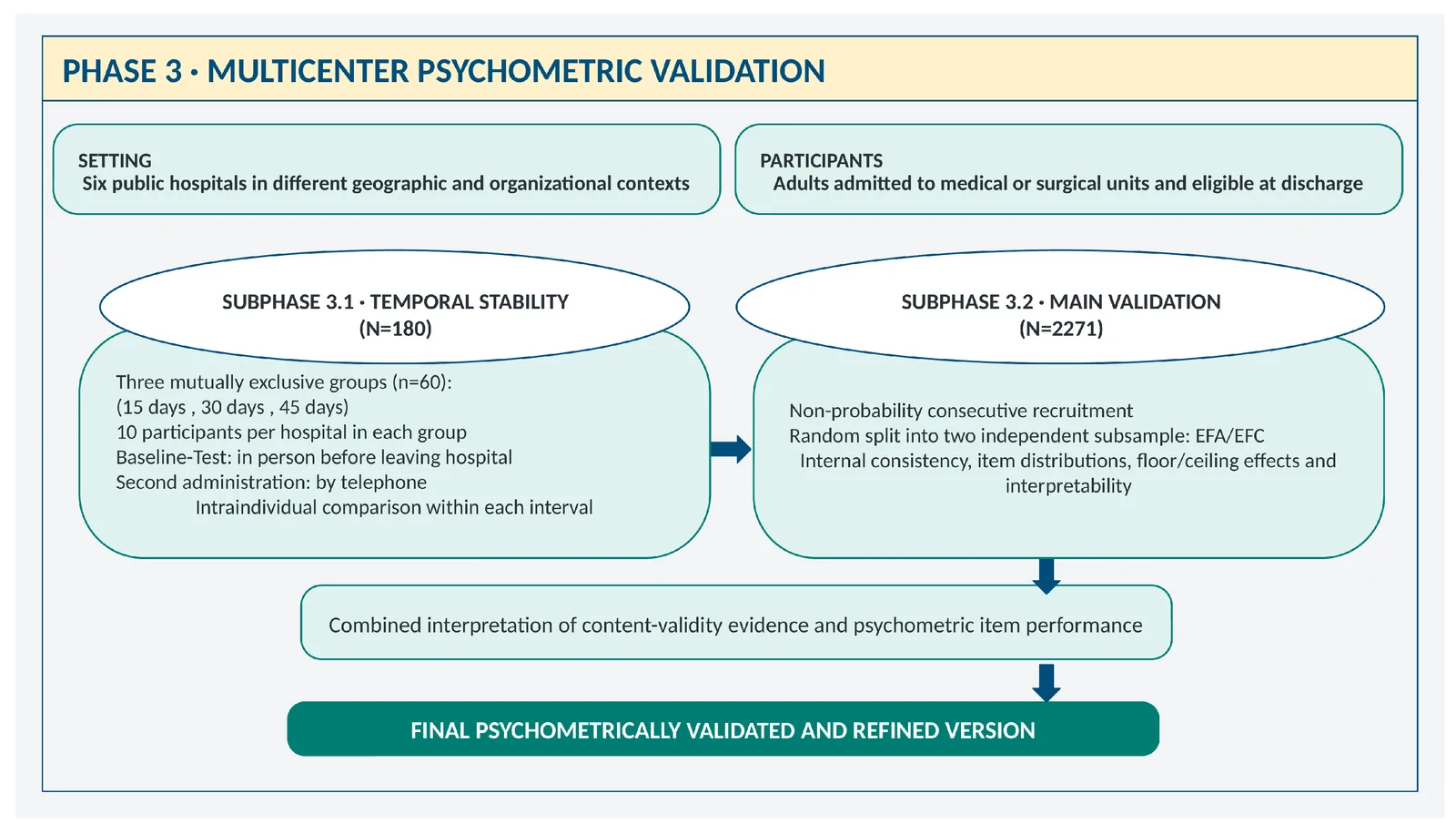
Diagram illustrating the third phase: multicenter psychometric validation of the provisional questionnaire. Arrows indicate the procedural flow.

A multicenter, observational, prospective study aimed at the psychometric validation of the provisional questionnaire (Figure 3).

This third phase will be divided into two subphases. The first will consist of a pilot study involving all collaborating hospital centers to assess the temporal stability and agreement of questionnaire responses between the first and second administrations within each of the predefined follow-up intervals of 15, 30 and 45 days. The second subphase will involve administering this provisional questionnaire to a sample of 2271 patients for subsequent validation.

*Sample*/*Participants*

Inclusion/exclusion criteria: Nonprobability consecutive sampling will be used until the required number of patients meeting the following inclusion criteria has been reached: patients who have had a hospital stay of more than 24 h; patients who have been informed that they will be discharged on the day the survey is administered, regardless of whether they have received their discharge documentation; patients hospitalized in medical or surgical units; and patients aged 18 years or older.

Patients who present with an unstable or serious clinical condition on arrival at the hospital that precludes an adequate assessment of their experience will be excluded, including those admitted in life-threatening emergency situations or with acute conditions that impair their ability to evaluate their experience. Patients with severe psychiatric disorders, cognitive impairment, or any condition that limits comprehension or the ability to communicate verbally or in writing will also be excluded. Cases involving language barriers that prevent correct completion of the instrument will likewise be excluded, as will patients who decline to participate or do not provide informed consent.

However, noncritically ill patients admitted through the emergency department will be included, provided that their clinical condition allows them to evaluate their care experience.

*Study Variables*

The primary variable will be patient-perceived experience in relation to the nursing care received during hospitalization, assessed using the final questionnaire obtained in the preceding phases.

Other variables will also be collected, including sex, age, marital status, educational level (no formal education, primary education, secondary education, vocational training, university education, upper secondary education, and other), employment status (student, employee, self-employed, retired, unemployed, and domestic worker), nationality, admitting specialty, type of admission (scheduled or emergency), type of hospital room (single or double), number of hospital admissions during the preceding year, admitting hospital, medical specialty responsible for the admission, and inpatient unit.

*Data Collection*

In both subphases, patients will be actively recruited through in-person visits to their rooms after medical discharge has been confirmed and before they leave the hospital. Before the questionnaire is administered, the research staff will inform patients why they are being invited to participate and explain the study objective. Participants will also receive an information sheet containing all relevant information, and any questions will be addressed. Patients will be informed that their responses will be confidential, and they will be required to sign the informed consent form before completing the questionnaire.

All participating patients will complete the questionnaire for the first time through an in-person, interviewer-administered assessment conducted in their room by nursing professionals who are members of the research team. These professionals will not have been directly involved in the care received by the patient during the hospital stay. The researcher will record the responses provided by patients in a digital case report form specifically designed for the study and accessible through a web domain. Limited literacy or difficulty reading the questionnaire will not, by themselves, constitute exclusion criteria, as the interviewer may read the items aloud and record the participant’s responses. Participants with sensory limitations may participate provided that they are able to understand the questions and communicate their responses using the standardized study procedure. No translated versions or ad hoc language interpretation will be used, as the psychometric validation will be conducted in Spanish. The data collection platform will be designed so that all questionnaire fields and all sociodemographic and clinical variables must be completed before a record can be finalized. It will also include format and range validation rules to reduce data entry errors. Incomplete records will remain in draft status until they have been completed appropriately, at which point their status will change to complete.

During the temporal stability subphase, the questionnaire will be administered for a second time by telephone at the assigned interval of 15, 30, or 45 days after hospital discharge. Responses will be recorded on the same digital platform.

Access to the platform will be restricted to the research team through personal, nontransferable passwords. The platform will be created and managed, and data security will be protected, by an external private company. This company will sign a confidentiality agreement in accordance with the regulations of the Instituto de Investigación Sanitaria de Aragón.

##### Subphase 3.1. Evaluation of Temporal Stability in a Multicenter Sample

The purpose of this subphase will be to obtain preliminary information on the temporal stability and agreement of the questionnaire before the main multicenter psychometric evaluation. No item elimination criteria will be applied on the basis of the results obtained in this pilot study.

*Sample*/*Participants*

A total of 180 patients discharged from inpatient units at the six participating hospitals will be included. Three independent, mutually exclusive groups will be established according to the planned interval before the second administration of the questionnaire, or retest (15, 30, or 45 days after discharge). Each group will comprise 60 patients, with 10 patients from each participating hospital. Patients in all three groups will complete the questionnaire at the time of medical discharge, before leaving the hospital, and again by telephone at the assigned interval. Participant will be allocated consecutively to de follow up groups of 15, 30 and 45 days according to a predefined rotating sequence (15 days → 30 days → 45 days), applied separately within each participating hospital.

The target sample of 60 participants per follow-up interval refers to participants with complete paired assessments. If a participant does not complete the second administration, the case will be recorded as lost to follow-up and will not be included in the temporal stability analysis. Recruitment will continue within each participating hospital until 10 participants with complete paired assessments have been obtained for each follow-up interval, resulting in 60 complete paired assessments per interval and 180 complete paired assessments overall. The number and proportion of participants lost to follow-up will be documented and reported.

The sample of 60 complete paired assessments per follow-up interval is intended to provide preliminary estimates of agreement and temporal stability rather than to perform direct comparisons between the three intervals. The precision of these estimates will be quantified using 95% confidence intervals.

The three intervals were selected a priori on a pragmatic basis to obtain preliminary information on response stability over progressively longer periods after discharge; they were not intended to establish a single optimal retest interval. Temporal stability will be examined through within-participant comparisons of the responses obtained in the two administrations for each of the three-time intervals. The three groups will be analyzed separately according to the follow up interval assigned for the second administration, and no direct comparisons between groups are planned.

##### Subphase 3.2. Multicenter Psychometric Validation in the Final Sample

*Sample*/*Participants*

The sample size was calculated separately for each participating hospital using the mean annual number of hospital discharges reported by each center for 2023, with a 95% confidence level and a 5% margin of error. A total planned sample size of 2271 patients was obtained by summing the separate calculations for each participating hospital. Therefore, the distribution of the total sample is not proportional to the centers’ discharge volumes.

Hospital El Bierzo reported a mean of 12,792 discharges and will contribute 373 participants; Hospital Universitari i Politècnic La Fe reported 30,297 discharges and will contribute 380 participants; Hospital Universitario La Paz reported 35,151 discharges and will contribute 381 participants; Hospital Clínico Universitario Lozano Blesa reported 26,300 discharges and will contribute 379 participants; Hospital Universitario Miguel Servet reported 36,000 discharges and will contribute 381 participants; and Hospital Universitari Sant Joan de Reus reported 18,500 discharges and will contribute 377 participants.

Although the total planned sample size was derived from the center-specific calculations described above, its adequacy for structural validity will be considered separately. The total validation sample will be randomly divided into two independent subsamples of 1136 and 1135 participants for EFA and CFA, respectively. According to COSMIN methodological criteria for structural validity [44], a factor-analysis sample size of at least seven times the number of items in the tested model and at least 100 participants is rated as very good. Using approximately 25 items as a provisional planning reference, based on the approximate length of the original ELIGES questionnaire and allowing for possible item additions or removals during the content-refinement and Delphi phases, each of the two planned factor-analysis subsamples substantially exceeds this criterion. The final number of items entering Phase 3 will depend on the results of Phases 1 and 2. Accordingly, the adequacy of each factor-analytic subsample will be evaluated against the actual number of items included in the version submitted to psychometric validation.

*Data Analysis*

The psychometric evaluation will be conducted within the Classical Test Theory framework. The specific analyses planned to examine item performance, structural validity, internal consistency, and temporal stability are described below.

Statistical analysis of the variables will be performed using Jamovi^®^, version 2.3.28 (The Jamovi Project, Sydney, Australia). Categorical variables will be presented as frequency distributions expressed as percentages for each category, whereas quantitative variables will be assessed for normality using goodness-of-fit tests and described using measures of central tendency and dispersion.

The data collection platform requires completion of all questionnaire fields and specific socioeconomic and clinical variables before a participant record can be finalized. Therefore, missing data are not expected in finalized records. Records that remain incomplete at the end of data collection will be counted but will not be included in the analysis.

With regard to content validity, during the modification of the questionnaire in Phases 1 and 2, expert patients will verify that the instrument includes items that are representative of the construct being measured and proportionate to the importance of its components, thereby providing evidence of content validity. To evaluate structural validity, the total validation sample of 2271 participants will be randomly divided in a 1:1 ratio into two independent subsamples: 1136 participants for exploratory factor analysis (EFA) and 1135 participants for confirmatory factor analysis (CFA). The first subsample will be used to identify the underlying dimensional structure, whereas the second will be used to evaluate the resulting factorial model using structural equation modeling.

Before the factor analyses are conducted, the distribution of responses to each item will be examined using frequencies and percentages for each response category. Highly skewed distributions and sparsely endorsed response categories will be identified and considered when evaluating the suitability and interpretation of the ordinal factor models. The proportions of responses in the lowest and highest categories of each item will be reported to assess potential item-level floor and ceiling effects. Once the final scoring structure has been established, floor and ceiling effects will also be examined for the total and subscale scores, where applicable, by reporting the proportions of participants obtaining the lowest and highest possible scores.

In the first stage, EFA will be performed in one of the independent subsamples using a polychoric correlation matrix, given the ordinal categorical nature of the responses to the questionnaire items. Factor extraction will be performed using the minimum residual method, with oblique Oblimin rotation to allow factors to be correlated.

Data adequacy will be evaluated using the Kaiser-Meyer-Olkin (KMO) index (expected value > 0.80) and Bartlett’s test of sphericity (*p* < 0.05). The number of factors will be determined primarily through parallel analysis, with eigenvalue criteria used as complementary information, while also considering the interpretability of the resulting factor solution. Factor loadings ≥ 0.40, communalities ≥ 0.30, and the absence of relevant cross-loadings will be considered acceptable criteria for item retention. A relevant cross-loading will be considered present when an item has loadings ≥ 0.30 on more than one factor. These cases will be assessed by considering both the magnitude of the loadings and the conceptual coherence of the item.

In the second stage, CFA will be performed in the other independent subsample using structural equation modeling with the robust weighted least squares mean and variance adjusted (WLSMV) estimator, appropriate for ordinal variables. Model fit will be evaluated using standard indices: Comparative Fit Index (CFI) and Tucker–Lewis Index (TLI) ≥ 0.90–0.95, Root Mean Square Error of Approximation (RMSEA) ≤ 0.06–0.08, and Standardized Root Mean Square Residual (SRMR) ≤ 0.08.

Modification indices and standardized residuals will be examined when model misfit is identified. Potential model modifications, including correlated residuals, will be considered only when supported by a theoretical or conceptual justification and not solely for the purpose of improving model-fit indices. If the CFA does not fully support the factorial structure identified by the EFA, the final factorial structure will be determined by considering the prespecified factor-analytic findings, including CFA model fit and item factor loadings, together with content-validity evidence and the conceptual relevance of the items. Any modification introduced after examination of the initial CFA results will be explicitly identified as exploratory.

The feasibility of assessing measurement invariance across the six participating hospitals will be evaluated as a secondary analysis once the final factorial structure has been established through the independent EFA and CFA subsamples. To maximize the stability of the multigroup estimates, this analysis will use the full Phase 3 sample (*n* = 2271), provided that the final factor structure, response distributions, and hospital-specific sample sizes allow adequate model estimation. Multigroup confirmatory factor analysis for ordinal data will be used when model estimation is feasible. Configural invariance will first be examined, followed by progressively constrained models assessing the equivalence of factor loadings and item thresholds across hospitals. Model comparisons will be based on changes in CFI, RMSEA, and SRMR. If full measurement invariance is not supported, partial measurement invariance will be considered by relaxing specific non-invariant parameters when statistically and conceptually justified, provided that adequate model estimation remains feasible.

Regarding temporal stability, agreement between responses obtained at hospital discharge and those collected by telephone will be evaluated separately for the follow-up intervals of 15, 30, and 45 days. Agreement at the item level will be assessed using weighted Cohen’s kappa coefficients, whereas agreement for total and subscale scores will be assessed using intraclass correlation coefficients, as appropriate. All estimates will be reported together with their 95% confidence intervals and interpreted as indicators of agreement and temporal stability between repeated administrations within follow-up interval. The precision of the estimates will be evaluated according to the width of these confidence intervals, and estimates with wide confidence intervals will be interpreted with caution. The three groups will be analyzed separately, and no direct comparisons between follow-up interval groups are planned.

Internal consistency will be evaluated for each identified dimension using Cronbach’s alpha and McDonald’s omega coefficients and for the overall score only if supported by the final factorial and scoring structure. Item discrimination indices will also be examined; decisions regarding item removal will consider these results together with factorial performance, content validity evidence, and the conceptual relevance of each item.

The baseline characteristics of the three patient groups corresponding to the follow-up intervals of 15, 30, and 45 days will be described and compared to identify relevant differences for interpreting the temporal stability results obtained.

The final composition of the questionnaire will be determined by considering both the evidence of content validity obtained during the first two phases and the psychometric performance of the items during Phase 3.

Once the psychometric validation of the instrument has been completed, an exploratory analysis will be conducted of the patient experience scores obtained and their potential relationships with the sociodemographic and clinical variables collected in the study. The analyses will be selected according to the nature and distribution of the variables and the final scoring structure of the validated instrument.

Given the multicenter design and the nesting of participants within the six participating hospitals, potential center-related differences will be explicitly considered. Hospital will be included as a categorical fixed effect or adjustment factor, as appropriate, in regression models examining associations between patient characteristics and questionnaire scores. Direct comparisons of questionnaire scores across hospitals will be undertaken only if the measurement invariance analysis supports sufficient comparability of the scores between centers. When such comparisons are appropriate, effect sizes and 95% confidence intervals will be reported. These analyses will be considered exploratory and will not be used to make claims of national representativeness.

## 3. Discussion

This article presents and describes in detail the methodological procedure followed for the content refinement and psychometric validation of the previously developed ELIGES-Patient Experience questionnaire, which assesses patient experience in relation to nursing care exclusively in the context of hospitalization. Incorporating the patient voice and responding to patients’ needs is a principle widely supported by the literature because of its direct association with improvements in healthcare quality. However, although patient experience has been considered a key element in the transformation of health systems internationally, challenges remain regarding its operational definition, its constituent dimensions, and how it can be integrated into genuinely person-centered models [4,14,15].

Historically, the measurement of patient experience has been limited both by variability in the term itself [1,16,53,54] and by the heterogeneity of the tools used for its assessment. This lack of homogeneity means that the different measurements are not comparable and considerably limits their use as tools for improving healthcare quality [14]. Therefore, the availability of specific, conceptually sound, and methodologically robust instruments is essential for advancing their practical applicability.

One of the most important distinguishing features of this protocol is the active involvement of patients in the questionnaire revision process, followed by multicenter psychometric validation. Their involvement throughout the methodological procedure is consistent with the principles of person-centered care and with evolving perspectives that promote stronger relationships between healthcare professionals and patients [2,3]. This involvement lends greater legitimacy to the instrument, facilitates the identification of dimensions that are genuinely meaningful to recipients of nursing care, and minimizes bias arising from an exclusively professional or organizational perspective [7,15]. Building on the initial validation of the ELIGES questionnaire, the present study incorporates systematic patient involvement in reviewing and revising the questionnaire content and extends its psychometric evaluation across six public hospitals in different geographic and organizational contexts.

Furthermore, the study uses a mixed-methods approach, integrating qualitative and quantitative techniques across the different phases of the validation process. The combination of these approaches makes it possible, on the one hand, to explore in depth the perceptions and meanings associated with patient experience and, on the other, to evaluate the measurement properties specified in this protocol through rigorous psychometric analyses. The literature emphasizes that mixed methods provide a broader and more contextualized understanding of complex health-related phenomena, particularly when the aim is to develop and validate instruments centered on patient-reported experiences of care. The integration of qualitative and quantitative data strengthens the methodological robustness of the study and improves the practical applicability of its results [55,56].

Following psychometric validation, the resulting tool may enable healthcare organizations and nursing professionals to identify specific areas for improvement by establishing monitoring indicators linked to the nursing care of patients admitted to inpatient units, directly and realistically [1,7].

In this way, the tool could help promote more individualized, safe, and person-centered care, while serving as a resource to support informed decision-making and the design of improvement policies aligned with value-based care models [6,57]. The usefulness of patient-reported information for identifying specific aspects of care processes and differences related to the care context has been highlighted in studies of patient experience [57]. Related evidence from studies of patient satisfaction has also shown that patients’ evaluations may vary according to organizational and care-context characteristics [58], although satisfaction and patient experience represent conceptually distinct constructs. In addition, the results obtained with the instrument may be used to analyze the relationship between patient experience and health outcomes, as well as the relationship between patient experience and the specific impact of nursing care on that experience [59].

## 4. Limitations

The nonprobability consecutive sampling of participants and the selection of the six participating hospitals may limit the generalizability of the results. Although the inclusion of centers from different geographic and organizational settings makes it possible to evaluate the instrument in diverse hospital contexts, the sample is not intended to be representative of all hospitals or of the hospitalized population in Spain. As with the application of any PREM, its application and the interpretation of its results in other settings should take into account the characteristics of the study population and the organizational context in which the instrument was validated.

The inclusion and exclusion criteria limit the applicability of the results to certain populations. The study will focus on patients aged 18 years or older who are admitted to medical and surgical units and will exclude, among others, patients with serious or unstable clinical conditions, severe psychiatric disorders, cognitive impairment, or language barriers that prevent completion of the instrument. Therefore, the results cannot be directly generalized to pediatric populations, critically ill patients, certain psychiatric populations, or individuals who cannot respond to the questionnaire in the language of administration. The findings may likewise not be directly generalizable to individuals whose sensory or communication limitations require adaptations beyond the standardized interviewer-administered procedure used in this study. Future research should evaluate the performance of the instrument in these settings and populations.

With regard to the qualitative phases, focus group members will be recruited through collaboration with the aforementioned associations, two of which focus on patients with cancer. This could limit the perspectives obtained to a more specific patient profile if sufficient variability is not achieved through the other participating associations.

Furthermore, the Delphi study will be conducted remotely through a digital platform, with telephone support from the research team when necessary. This strategy is intended to resolve any technical or operational questions in real time, minimizing the possibility that technology becomes a barrier to participation. These measures are intended to reduce potential participation difficulties among individuals with lower digital literacy or less routine access to technological resources.

During the psychometric validation phase, in-person administration of the questionnaire immediately before hospital discharge may influence responses because of the context in which the assessment is conducted. Because the questionnaire will be interviewer-administered by nursing professionals who are members of the research team, social desirability or interviewer bias may arise, with a tendency to provide more favorable responses regarding the care received. To reduce this influence, the questionnaire will be administered by professionals who were not directly involved in the care received by the patient. However, this measure does not completely eliminate the potential effect associated with the interviewer’s professional role and the discharge context. These potential influences will therefore be considered when interpreting the patient-experience responses obtained.

With regard to the temporal stability assessment, the established time intervals may involve reinterpretation or recall of the patient’s experience, particularly at the longer intervals. Because the first administration will be conducted in person and the second by telephone, a potential effect associated with the mode of administration cannot be ruled out. Loss to follow-up may occur between the first and second administrations. Although recruitment will continue until 60 participants with complete paired assessments are obtained for each follow up interval, loss to follow up may still introduce differences between participants who complete both administrations and those who do not. Therefore, the observed agreement cannot be interpreted as an isolated estimate of test–retest reliability, because variability may also reflect elapsed time, recall, reinterpretation of the hospitalization experience, or mode of administration effects.

Finally, obtaining evidence on an instrument’s measurement properties is a cumulative process. The psychometric scope of this protocol is limited to content and structural validity, internal consistency, temporal stability and interpretability. Criterion validity will not be assessed because the study does not include an appropriate reference standard for patient experience specifically related to nursing care during hospitalization. Once the questionnaire’s structure has been established, this evidence may be expanded in future studies through analyses of convergent and discriminant validity using conceptually appropriate external measures, and through known-groups validity based on specific, theoretically grounded, prespecified hypotheses regarding the expected differences.

## Data Availability

No new data were created or analyzed in this study. Data sharing is not applicable.

## Public Involvement Statement

Expert patients will collaborate in the development of Phases 1 and 2 of this study. Patient associations throughout Spain will be contacted for recruitment.

## Guidelines and Standards Statement

The methodological approach to content refinement and psychometric validation of the questionnaire was based on internationally accepted recommendations for the development and evaluation of health measurement instruments, including COSMIN methodology where applicable.

## Abbreviations

The following abbreviations are used in this manuscript:

AMACGEMA	Association of Aragonese Women with Genital and Breast Cancer
AECC	Spanish Association Against Cancer
CFI	Comparative Fit Index
CFA	Confirmatory factor analysis
CAHPS	Consumer Assessment of Healthcare Providers and Systems
EFA	Exploratory factor analysis
EUROPEP	European Task Force on Patient Evaluations of General Practice Care
ALCER	National Federation of Associations for the Fight Against Kidney Diseases
HCAHPS	Hospital Consumer Assessment of Healthcare Providers and Systems
KMO	Kaiser–Meyer–Olkin index
NHS	National Health Service
NICE	National Institute for Health and Care Excellence
PPIC	Patient Perceptions of Integrated Care
PCMH	Patient-Centered Medical Home
RMSEA	Root Mean Square Error of Approximation
SRMR	Standardized Root Mean Square Residual
TLI	Tucker–Lewis Index
WHO	World Health Organization
